# The Hepatitis C Virus E1 Glycoprotein Undergoes Productive Folding but Accelerated Degradation When Expressed as an Individual Subunit in CHO Cells

**DOI:** 10.1371/journal.pone.0023838

**Published:** 2011-08-17

**Authors:** Valentina Botti, Alessia Bianchi, Steven K. H. Foung, Marcello Merola

**Affiliations:** 1 Novartis Vaccines and Diagnostics, Siena, Italy; 2 Department of Pathology, Stanford University, Stanford, California, United States of America; 3 Department of Structural and Functional Biology, University of Naples “Federico II”, Naples, Italy; University of Minnesota, United States of America

## Abstract

Hepatitis C Virus E1E2 heterodimers are components of the viral spike. Although there is a general agreement on the necessity of the co-expression of both E1 and E2 on a single coding unit for their productive folding and assembly, in a previous study using an in vitro system we obtained strong indications that E1 can achieve folding in absence of E2. Here, we have studied the folding pathway of unescorted E1 from stably expressing CHO cells, compared to the folding observed in presence of the E2 protein. A DTT-resistant conformation is achieved by E1 in both situations, consistent with the presence of an E2-independent oxidative pathway. However, while the E1E2 heterodimer is stable inside cells, E1 expressed alone is degraded within a few hours. On the other hand, the oxidation and stability of individually expressed E2 subunits is dependent on E1 co-expression. These data are consistent with E1 and E2 assisting each other for correct folding via different mechanisms: E2 assists E1 by stabilizing a semi-native conformation meanwhile E1 drives E2 towards a productive folding pathway.

## Introduction

The Hepatitis C virus (HCV) is the etiologic agent of an important global disease causing chronic liver infection, which can lead to cirrhosis and hepatocelllular carcinoma [1]. HCV shares common features with pestiviruses and flaviviruses, such as being enveloped and consisting of single stranded positive RNA genome coding for a single open reading frame (ORF), but has been classified within a separate genus of the *flaviviridae* family [2]. The mature HCV viral proteins are generated via co- and post-translational cleavages that are dependent on the concerted action of host and viral proteases. The 5′ end of the genome encodes for the structural proteins: Core, the unique proteic component of the viral nucleocapsid, and two glycoproteins, E1 and E2, responsible for viral attachment and entry into host cells [3], [4], [5]. Intracellularly expressed E1 and E2 lead to the formation of non-covalent associated heterodimeric complexes. E2 is incompletely cleaved from the adjacent p7 protein generating a detectable E2p7 product whose role in viral particle formation, if any, is still unknown. The remaining two thirds of the genome encodes the non-structural (NS) proteins NS2, NS3, NS4A, NS4B, NS5A and NS5B [2]. Although NS2 is dispensable for replication, it has been classified as a non-structural protein since it has not been found to be assembled into virus particles, even though it is involved in viral assembly [6], [7].

Establishment of a functional non-covalent E1E2 heterodimer is a crucial step for viral particle formation. During translation of the polyprotein, appropriate signal sequences target the two glycoproteins to the endoplasmic reticulum (ER) where they are released from the polyprotein by the action of the host signal peptidase. This ER enzyme is oriented in the lumen and cleaves the Core-E1, E1-E2 and E2-p7 junctions [8], [9]. In the ER, HCV envelope proteins acquire 4-5 and 11 N-linked glycosylation chains for E1 and E2, respectively, and remain anchored to the membrane through their hydrophobic C-terminal domains. It has been reported that these transmembrane regions carry crucial determinants for ER retention and E1E2 heterodimerization [10]. Formation of the heterodimer is a slow process that requires up to 6 hours to be completed [8], [11].

A substantial number of reports have analyzed the folding/assembly of HCV structural proteins and, in particular, individually expressed E2. There are several reasons for the increased interest in the E2 glycoprotein. Firstly, E2 directly contacts host membrane proteins required for virus entry, including CD81 [12] and SR-B1 to which direct binding has been proven with the soluble E2 protein [13]. Secondly, E2 is the target for most of the neutralizing antibodies generated in mice or isolated from HCV infected patients [14], [15]. Thirdly, individually expressed E2, as well as truncated forms of this protein, have been found to properly fold and generate epitopes recognized by conformational antibodies [16]. Indeed, a truncated form of this protein, that is soluble and easier to purify than the full-length protein, has also been indicated as a vaccine candidate [17].

Although E2 represents an appealing target for the development of an anti-HCV prophylactic vaccine, recent trials suggest that the administration of both HCV glycoproteins as a heterodimer is needed [18], [19]. The current view is that co-expression of E1 and E2 is required for the folding/assembly of E2 in its native structure (reviewed in [20]). A purified soluble truncated form of E1 was also investigated as a therapeutic vaccine in a pilot study, but no significant reduction of HCV infection was observed [21]. Thus, although the presence of anti-E1 neutralizing antibodies has been described [22], [23], formation of the heterodimeric complex seems to be strictly required to generate an immunoprotective antigen. Folding analysis of unescorted E1 has received less attention as it was reported to be unable to fold properly when expressed in the absence of E2 [16]. However, in a previous study we observed that the oxidation process and the transient association with the ER chaperone calnexin of individually expressed E1 proceeded as expected for a correctly folded protein [24]. These data were obtained using an *in vitro* translation system that did not allow us to follow the fate of E1 at late time points post-synthesis. Thus, although E1 appeared to reach a chaperon released conformation, we could not analyze its stability as single expressed protein.

We sought to verify whether E1 can achieve a native conformation in absence of E2 and generate a stable species to be purified and tested for immunological protection. To overcome the limitation of the *in vitro* system, in this study we analyzed the oxidative folding of E1 individually expressed in stably transfected CHO cells and compared the same process when the glycoprotein is expressed together with E2 as a single coding unit. In CHO cells, the oxidation and folding pathway of E1 is identical to the one observed for the polyprotein, except for an accelerated kinetics. The folding process of individually expressed E2 (expressed as E2p7 precursor) was also analyzed. In the absence of E1, only a low amount of properly oxidized E2 could be seen suggesting that misfolding pathways predominate in these conditions. However, both E1 and E2 expressed as single proteins were not stable in the cells and they were cleared within a few hours after reaching a conformation that was resistant to the reducing agent dithiothreitol (DTT).

## Results

### Experimental background

**Figure 1:**
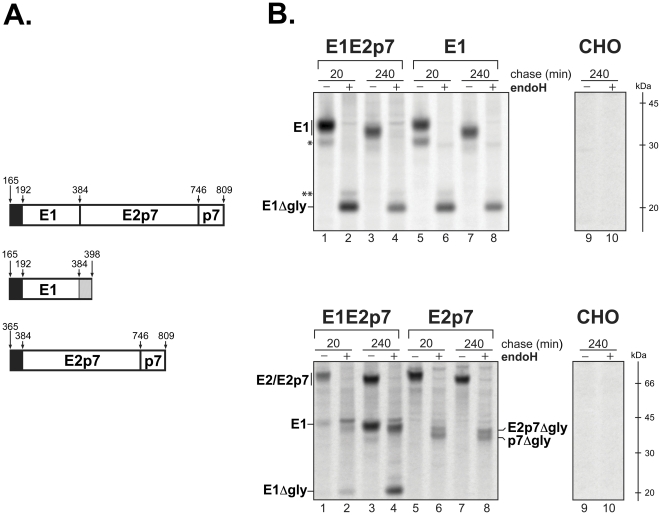
Endoglycosidase H sensitivity of HCV glycoproteins E1 and E2p7. (A) Recombinant constructs for E1, E2p7 and E1E2p7 expression in CHO cells. Sequences coding for the structural protein E1 (aa. 165–398), E2p7 (aa. 365–809) and E1E2p7 (aa. 165–809) were amplified from a replicon containing the entire HCV genome of HCV genotype 1b, subtype ConI as described in the Materials and Methods. These constructs were used to stably express the proteins in CHO cells. Black segments represent the E1 (aa. 165–191) and E2 (aa. 366–384) signal sequences. The N-terminus 15 amino acids of the E2 protein are shown as a dashed segment in the E1 construct. (B) CHO cells expressing E1, E2p7 or E1E2p7 were pulse-labeled with [^35^S]-methionine and -cysteine for 20 min and chased for the indicated time periods. PNSs were immunoprecipitated with an anti-E1 (top panel) or anti-E2 antibody (lower panel) and further treated with (+) or without (−) EndoH. Proteins were separated on 10% SDS-PAGE in reducing conditions. Asterisks indicate non-specific bands. E1ΔGly indicates the E1 protein co-immunoprecipitated with the anti-E2 antibody and subsequentlydeglycosylated by EndoH treatment.

In this study we have used the structural region of the hepatitis C virus Con1 strain (1b) for all the constructs [25] (EMBL database accession number AJ238799). The coding regions corresponding to E1E2p7, E1 and E2p7 have been amplified and cloned into an eukaryotic expression vector. The E1 protein contains an additional 15 amino acids C-terminus belonging to the N-terminus sequence of E2. The presence of this region is required to assure the correct cleavage and glycosylation of E1, as reported previously [24]. These constructs are shown in figure 1A. Constitutive expression of E1E2p7, E1 and E2 in CHO has been achieved using stable transfected cell lines standard procedure (detailed in Material and Methods). The corresponding cell lines constitutively expressing E1E2p7, E1 or E2p7 (CHO-E1E2p7, CHO-E1 and CHO-E2p7, respectively) were screened by Western blot analysis and immunoprecipitation following metabolic ^35^S-Met/Cys labeling (data not shown). One clone of each construct was selected on the basis of the amount and stability of expressed proteins after several cell passages. The folding kinetics of the products expressed by CHO-E1E2p7 was used as an internal control to evaluate the folding of E1 or E2p7 expressed alone in CHO cells.

We have previously shown that the chimpanzee antiserum Ch-L559 was able to recognize all conformational species of E1 and E2/E2p7 from strain HCV-1a [8], [26]. Unfortunately this tool failed to reveal HCV proteins from strain HCV-1b in immunoprecipitation experiments, whereas it was still useful for Western blot analysis of E2/E2p7. Thus, to perform our folding analysis we have used human monoclonal antibodies generated from B-cells isolated from HCV infected patients [27]. In particular, we screened 9 anti-E2 monoclonal antibodies (mAb), five of which recognized E2, with almost the same specificity. Among these we selected the mAb CBH-7 that recognized a DTT-sensitive conformational epitope of the E2 protein. Concerning E1, we used the CBH-111 mAb that recognizes both reduced and oxidized forms of E1 [22]. This antibody binds a conserved linear epitope close to the N-terminus of E1 but, in our hands, does not lead to E2 co-immunoprecipitation.

Oxidative kinetics is visualized by SDS-PAGE electrophoretic mobility differences of partially or fully oxidized proteins under non-reducing conditions, compared to the identical sample migrated in reducing conditions [28]. We will refer to samples as i) reduced proteins (**red**) for those in which DTT has been added immediately prior to electrophoretic analysis, ii) oxidized (**ox**) species for those that migrates faster than the reduced counterpart and iii) unoxidized (**unox**) for those that display slower migrating species in non-reducing conditions, whose intra-chain disulfide bonds are variable.

### EndoH digestion

Our aim was to study the folding capability and stability of HCV glycoproteins expressed alone in eukaryotic cells. In particular we meant to focus on the E1 protein considering that this topic has been poorly addressed especially in the absence of the other major HCV glycoprotein E2.

As a first step, we performed an Endoglycosidase H (EndoH) digestion to establish whether unescorted E1 was retained in the ER. This enzyme hydrolyzes high mannose N-linked chains resident in the ER but it does not act on carbohydrate structures further modified in the Golgi. The EndoH sensitivity of the sugar moiety of glycoproteins represents an easy way to distinguish between ER-localized proteins and species that have entered the anterograde trafficking pathway. EndoH digestion analysis of protein expressed by the E1 and E2p7 constructs compared with the control E1E2p7 was performed at 20 min and 4 hrs chase-points.

The top panel of figure 1B shows the result of the analysis performed on CHO-E1 and CHO-E1E2p7 cells. In both samples the behavior of the E1 glycoprotein was identical at both times post-synthesis. On the contrary, as shown in the bottom panel of figure 1B, two major differences were revealed for E2 expressed alone, with respect to the control: i) the relative amount of E2 and E2p7 species and ii) the faster migration of deglycosylated E2/E2p7 in CHO-E2p7 cells with respect to the control sample (lanes 6 and 8). Following deglycosylation, E2 and E2p7 were clearly separated on the gel and, for products expressed by CHO-E1E2p7, the cleavage of the precursor was time dependent (lanes 2 and 4). Indeed, comparing the relative intensity of E2 and E2p7 bands from CHO-E2p7 cells, revealed that the amount of mature E2 did not substantially change between 20 min and 4 hours of chase (lane 6 and 8). In the E2p7 sample we did not observe the progressive maturation of the E2p7 precursor since the percentage of the precursor appeared to be roughly equal irrespective of the chase-time. Concerning the faster migration of the deglycosylated E2 and E2p7 observed in CHO-E2p7 cells, the most likely explanation would be a further proteolytic cleavage at the N-terminal of E2 expressed singularly, but we did not investigate this possibility further.

In conclusion, our data show that E1 expressed alone is correctly matured by the signal peptidase and subjected to proper ER-type glycosylation. On the other hand, E2/E2p7 stably expressed in CHO-E2p7 cells shows remarkable differences compared with the same species from CHO-E1E2p7 cells.

### Oxidative kinetics

**Figure 2:**
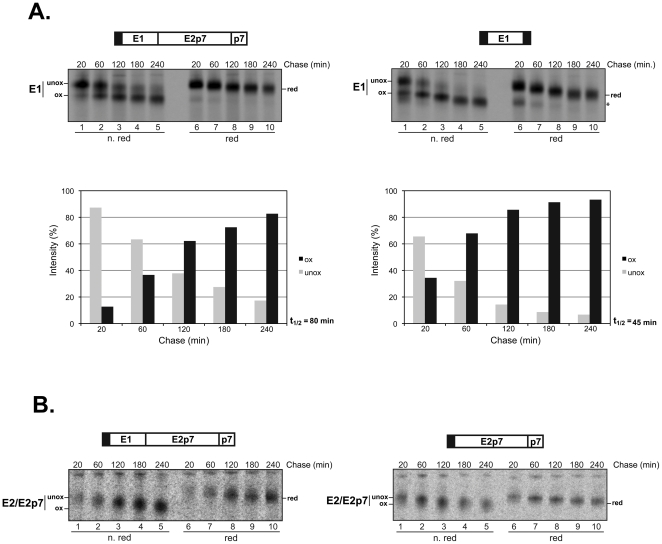
Oxidation kinetics of E1 and E2p7 HCV glycoprotein. CHO cells stably transfected with constructs expressing E1, E2p7 or E1E2p7 were pulse-labeled with [^35^S]-methionine and -cysteine for 20 min and chased for the indicated time-periods. PNSs were immunoprecipitated with (A) CBH-111 anti-E1 monoclonal antibody or (B) CBH-7 anti-E2 monoclonal antibody. Samples were analyzed on 10% SDS-PAGE in non-reducing (lanes 1–5) and reducing (lanes 6–10) conditions. For E1, the relative intensity of the unoxidized (grey bars) and oxidized (black bars) forms were quantified for the different chase-times. The E1E2p7 wild type species is reported on the left graph, E1 alone on the right graph. The half-time (t_1/2_) of E1 oxidation represents the chase-time at which half of the protein is in its oxidized form. Symbols refer to: ***ox*** for oxidized; ***unox*** for unoxidized; ***red*** for reduced.

Pulse-chase experiments from 20 min to 4 hrs were performed to study the oxidation kinetics of E1 and E2/E2p7 in stably transfected CHO cells. The pulse-chased post-nuclear supernatants (PNSs) from CHO-E1E2p7, CHO-E1 and CHO-E2p7 cells were subjected to immunoprecipitation, SDS-PAGE analysis and autoradiography. Results from representative gels are shown in figure 2. Panel A shows the oxidative kinetics of E1 synthesized in the presence of E2 (left) or in its absence (right). The time-dependent oxidation of E1 was followed and quantified by the appearance of its faster migrating band (ox- in the figure). As expected, the relative intensity of this band increased over time until reaching a plateau. This process is common to both constructs but with different kinetics. To better visualize such differences, on the bottom of figure 2A a densitometric analysis of the gels for each chase-time is shown, reporting the intensity of either the oxidized or unoxidized forms expressed as a relative percentage. The E1 protein from CHO-E1E2p7 cells showed a half-time (t_1/2_) of oxidation of about 80 min, still increasing after 4 hrs post-synthesis. In contrast, E1 expressed alone oxidized faster, with a t_1/2_ value of about 45 min, reaching the plateau within 3 hrs of chase.

In figure 2B a representative experiment of the analogous oxidative analysis of CHO-E2p7 compared to the control CHO-E1E2p7 cell line is shown. Since the anti-E2 CBH-7 antibody used for the immunoprecipitations recognizes only conformational epitopes, the unoxidized forms of E2 and E2p7 were not present on the gel. While this limitation does not allow a detailed analysis, such as densitometry of the different species, the significant features of the oxidation pathway of E2/E2p7 expressed individually can still be observed. E2 from the control cells showed a time dependent migration shift over the 4 hrs chase-period (figure 2B, left panel). In contrast, E2 expressed in the absence of E1 revealed a less pronounced shift between non-reduced and reduced samples, as well as faster oxidative kinetics reaching the plateau within 1 hr post-synthesis (figure 2B, right panel. The position of the band in lane 3, 4 and 5 does not change. And the intensity of the bands is better resolved in reducing conditions, lanes 6–10). Preliminary conclusions from this analysis are consistent with an accelerated but regular oxidation pathway for E1 in absence of the major HCV glycoprotein. However, E2/E2p7 oxidation appeared to be severely impaired.

### DTT-resistance analysis

**Figure 3:**
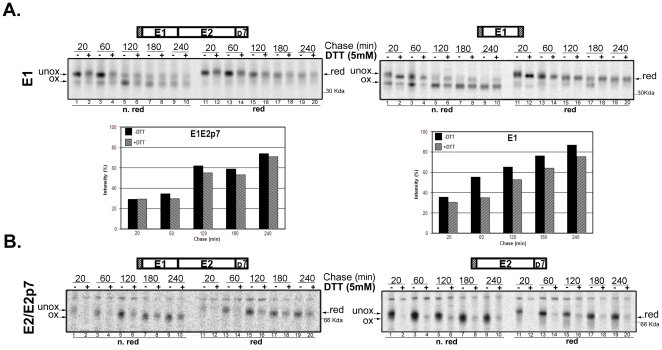
Analysis of DTT-resistance of HCV proteins E1 and E2. (A) CHO cells stably transfected with E1E2p7 or E1 were pulse-labeled with [^35^S]-methionine and –cysteine for 20 min and chased for different time-periods in the presence (+) or absence (−) of 5 mM DTT added exogenously 5 min before the indicated chase-time. PNSs were immunoprecipitated with the CBH-111 anti-E1 antibody and samples analyzed on 10% SDS-PAGE in non-reducing (lanes 1–10) and reducing (lanes 11–20) conditions. Graphs below represent the percentage of oxidized E1 present in samples derived from untreated (black bars) and DTT-treated (dashed bars) cells. The relative intensity-percentage is calculated by densitometric analysis of the unoxidized and oxidized forms in non-reducing conditions in the presence (lanes 1, 3, 5, 7, 9) or absence (lanes 2, 4, 6, 8) of 5 mM DTT added exogenously 5 min before the indicated chase-period. Left graph reports values obtained when expressed with E2p7, right graph when expressed individually. (B) CHO cells stably transfected with E1E2p7 or E2p7 were pulse-labeled with [^35^S]-methionine and –cysteine for 20 min and chased for different time-periods in the presence (+) or absence (−) of 5 mM DTT added exogenously 5 min before the indicated chase-time. PNSs were immunoprecipitated with CBH-7 anti-E2 antibody and samples analyzed on 10% SDS-PAGE in non-reducing (lanes 1–5) and reducing (lanes 6–10) conditions. Symbols refer to: ***ox*** for oxidized; ***unox*** for unoxidized; ***red*** for reduced.

Proteins stabilized by intra-chain disulfide bonds are usually resistant to reducing agents when they reach native conformation. On the contrary, folding intermediates are fully or partially susceptible to such chemicals [28]. This property is useful to judge the oxidation status of a conformer. By adding an exogenous reducing agent to a mixed population of folding protein, the native species does not modify its migration pattern on a non-reducing SDS-PAGE. On the other hand, folding intermediates will be partially or fully reduced, migrating substantially slower in the same electrophoretic conditions. Figure 3 shows a representative experiment of DTT resistance of CHO-E1E2p7, CHO-E1 and CHO-E2p7 cell lines. The antibodies used for immunoprecipitation were anti-E1 CBH-111 (figure 3A) and anti-E2 CBH-7 (figure 3B).

Fig. 3A shows the pattern of E1 sensitivity to the exogenously added reducing agent, both expressed in presence (left) or absence (right) of E2. This parameter was evaluated on the basis of SDS-PAGE migration in non-reducing conditions by comparing samples derived from untreated and DTT-treated cells. Quantification by densitometric analysis of the E1oxidized fraction in these two conditions is reported in the graphics at the bottom of figure 3A. This representation allows a direct comparison of the behavior of the glycoprotein expressed by the two constructs. Both in absence and in presence of E2, the amount of DTT-resistant species increased over the entire chase-period. Individually expressed E1 shows an accelerated oxidation pathway, consistent with the kinetics shown in figure 2, while the kinetics of the DTT-resistant conformation appeared similar between the two samples (figure 3A). Indeed, at the end of the chase-period, the percentage of the DTT-resistant conformer relative to the total E1 was comparable among the two constructs (graphic in figure 3A). This result suggests that E1 acquires a tighter conformation independently of E2 co-expression.

Once again, substantial differences in the E2 DTT resistance pathway have been revealed by analysis of E2 expressed alone, as shown in figure 3B. To be able to compare different samples, we normalized the PNSs submitted to immunoprecipitation. This procedure was necessary since the anti-E2 antibody used in this study did not recognize unoxidized forms of the protein, thus a densitometric analysis on the two conformers (*ox* versus *unox*) of the same protein could not be performed. To assess the percentage of E2 that acquired DTT-resistance, following cell lysis and pre-clearing of the PNSs, an equivalent amount of radiolabeled proteins (measured as TCA insoluble radioactive fraction) were submitted to immunoprecipitation [8]. The amount of the DTT-resistant fraction could therefore be estimated from the total immunoprecipitated pool by comparing the intensity of the bands obtained in the presence and absence of exogenously added DTT.

A representative experiment of DTT-resistance by E2/E2p7 expressed in CHO-E1E2p7 cells is shown in the left panel of figure 3B. It can be observed that both the oxidized (lanes 1, 3, 5 and 7) and DTT-resistant (lanes 2, 4, 6 and 8) E2 species increased over time, but did not reach a plateau within the 4 hours of chase. On the other hand, E2/E2p7 expressed in the absence of E1 (figure 3B, right panel) showed an increased amount of the DTT resistant species within 1 hr of chase (compare lanes 2 and 4) that did not rise further over the 4 hrs chase-period (lane 4 compared to 6, 8 and 10). Thus, oxidation and achievement of a DTT resistant conformation by E2/E2p7 expressed alone was faster but much less efficient than when expressed together with E1.

Taken together these experiments confirmed that the achievement of E1 fully oxidized conformation is independent of the presence of E2, whereas the requirement of E2 for its glycoprotein partner is crucial for its correct oxidative folding.

### HCV glycoprotein stability in CHO-E1E2p7, CHO-E1 and CHO-E2p7

**Figure 4:**
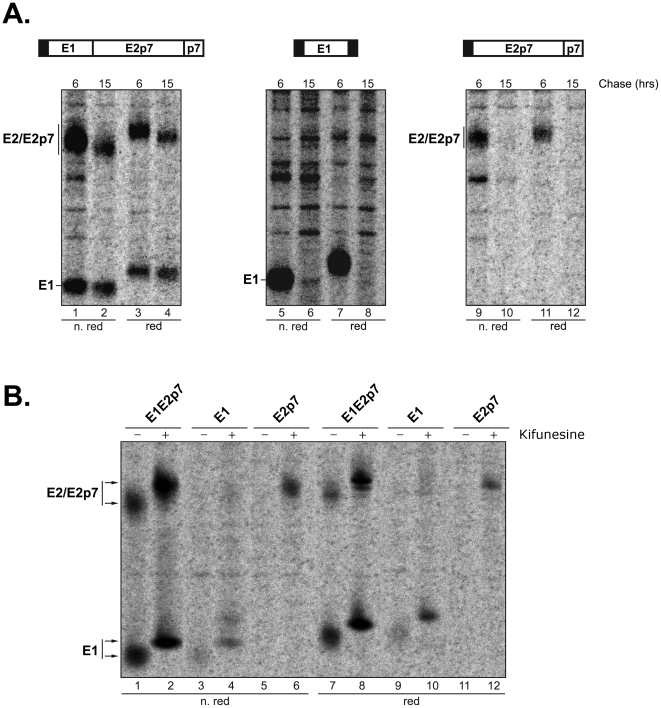
E1 and E2 stability in CHO stably trasfected cells. (A) CHO cells expressing E1, E2p7 or E1E2p7 were pulse-labeled with [^35^S]-methionine and –cysteine for 20 min and chased for 6 and 15 hrs. PNSs were immunoprecipitated with the CBH-111 anti-E1 monoclonal antibody (central panel) or the CBH-7 anti-E2 monoclonal antibody (lateral panels). Samples were analyzed on 10% SDS-PAGE in non-reducing (lanes 1, 2, 5, 6, 9, 10) or reducing (lanes 3, 4, 7, 8, 11, 12) conditions. (B) CHO cells stably transfected with E1, E2p7 or E1E2p7 were pulse-labeled with [^35^S]-methionine and –cysteine for 20 min and 15 hrs. During the pulse- and chase-time, the medium was supplemented with (+) or without (−) 20 µM kifunesine. PNSs were immunoprecipitated with the CBH-111 anti-E1 antibody (lanes 3, 4 and 9, 10) or the CBH-7 anti-E2 monoclonal antibody (lanes 1, 2, 5, 6, 7, 8, 11, 12). Samples were analyzed on 10% SDS-PAGE in non-reducing (lanes 1–6) or reducing (lanes 7–12) conditions.

Recombinant HCV glycoproteins synthesized in stably transfected eukaryotic cells are quite stable products that, once associated as native heterodimers, accumulate in the cells. The experiments described in the above paragraphs suggested that E1 independently achieves proper folding, while only a minor fraction of the total E2 expressed alone reaches a fully oxidized and supposedly native conformation. We sought to verify the stability of individually expressed E1 and E2 in our system compared to the stability of the same proteins expressed on a single coding unit. To this aim we performed several experiments extending the chase-period to 6 and 15 hrs. To be able to compare samples at different chase-periods, we immunoprecipitated equivalent TCA insoluble counts as described above. The left panel of figure 4A shows the results obtained with cells transfected with the reference constructs E1E2p7, the PNS of which was immunoprecipitated with the anti-E2 CBH-7 mAb. The amount of E1E2 heterodimer decreased from 6 to 15 hrs (lane 2 compared to lane 1) even though the two bands remained clearly detectable. The middle panel shows E1 immunoprecipitated with the CBH-111 anti-E1 antibodies from CHO-E1 PNS. Unequivocally, at the 15 hrs chase-period the E1 band disappeared almost completely (lane 6 or 8). Identical results were obtained from analysis of E2p7 expressed alone and immunoprecipitated with CBH-7, shown in the right panel. At 6 hours we observed the plateau of E2 accumulation but it was completely cleared by 15 hrs post-synthesis (lanes 10 or 12). Post-synthetic accumulation at 6 and 15 hours represents the two chase-periods that better embody our results. After 6 hrs chase, in fact, both proteins from all three constructs reached the maximum amount (data not shown). Similarly, we choose 15 hrs chase-time since intermediate chase-periods resulted in variable percentages of degradation. These data strongly indicate that both HCV glycoproteins, when expressed as isolated subunits, are not stable inside the cells.

The next experiment was designed to establish whether the proteasome was implicated in E1 and E2/E2p7 degradation. However, we could not use direct proteasome inhibitors, such as lacticystin or MG132, since prolonged incubation with these compounds led to extensive cell death. To overcome this problem we used kifunesine, an ER mannosidase I inhibitor [29]. Misfolded glycoproteins targeted for degradation need to be retro-translocated into the cytosol, a process that requires recognition of the oligosaccharide moiety by a specific family of receptors named EDEM [30], [31]. The EDEM substrate is generated by the action of the ER mannosidase I, a slow acting enzyme which selectively removes a single α-mannose from the core-glycan of glycoproteins unable to escape the ER quality control machinery [32]. Thus, inhibition of mannosidase activity acts upstream of proteasome degradation by blocking the access route to the cytosol where the ubiquitin-proteasome system is localized.

CHO-E1, CHO-E2p7 and CHO-E1E2p7 cells were pre-incubated for 30 min with kifunesine, which was also maintained in the culture media during pulse and the 6 and 15 hr chase-time. At the end of these periods, cells lysates were immunoprecipitated and HCV E1 and E2 proteins analyzed on SDS-PAGE. The results from this experiment are shown in figure 4B. In the CHO-E1E2p7 control sample, E1 and E2 bands are still present in a significant amount in absence of kifunesine with a small increase in the presence of the drug (lanes 1 and 7 compared to lanes 2 and 8). The apparent MW of both proteins is shifted higher in the drug-treated samples, as expected after the inhibition of trimming of high mannose structures. E1 and E2 from CHO-E1 and CHO-E2p7 lysates, respectively, are detectable at a low amount in samples derived from kifunesine treated cells (lanes 4, 10 and 6, 12). Thus, the presence of the inhibitor is able to trigger a certain degree of protection from degradation. This result is consistent with the quick degradation of E1 and E2 when they are not co-expressed and is consistent with the involvement of the proteasomal pathway in the clearance of the HCV glycoproteins.

## Discussion

During infection of host cells by enveloped virus, the fusion of the viral envelope and the cellular membrane is facilitated by viral fusion proteins. The fusion mechanism shares common features in all viral families so far described, but the overall structure of the different fusion proteins evolved separately and they are characterized as three distinct classes [33]. Fusion proteins of the *Togaviridae* and *Flaviviridae* families belong to the class II [34], [35], which most likely also contains the E2 protein of HCV [36]. Class II fusion proteins share a common molecular architecture and need a “partner” subunit with which they fold and form heterodimers. The chaperone-like role of this second protein, whose synthesis precedes that of the class II proteins, has been demonstrated for Semliki Forest virus (SFV) and tick-borne encephalitis viruses (TBE) [37], [38]. Another aspect shared by these chaperone-like subunits is their ability to fold correctly once expressed individually. Indeed, both the p62 protein of SFV and the prM protein of TBE did not show any major difference in folding once expressed in the absence of the second subunit on the coding unit [37], [38]. Differently from both SFV p62 and TBE prM proteins, however, E1 does not undergo proteolytic processing prior to virus release. Furthermore, the resulted HCV infectious particles are resistant to acidic pH treatment [39]. These data may suggest a different role of E1 during entry or fusion steps.

Previous data generated in our laboratory suggest that the HCV glycoprotein E1 could play a role analogous to p62 and PrM in viral maturation. In an *in vitro* translation system, unescorted E1 was able to achieve a complete folding process once provided with a small region of E2 that ensured the correct topology of the C-terminus [24]. Afterwards, folding/assembly analysis of E1E2 stably expressed in eukaryotic cells revealed that E1 oxidation preceded E2 maturation, a behavior consistent with a potential chaperone-like role for the E1 protein [8]. The analysis performed here extends these previous reports by analyzing the mechanism of unescorted E1 folding in stable transfected CHO cells and its stability at longer time points post-synthesis. The folding pathways of individually expressed E2, and E1E2 as single coding unit, were evaluated in the same system.

In terms of its general features, the individually expressed E1 was indistinguishable from the E1 expressed in the control expression unit: it did not present high molecular weight aggregates, it received the proper number of carbohydrates chains and was retained in the ER. The oxidative kinetics of E1 expressed as a single protein showed a complete oxidation between 2 and 3 hours post-synthesis (t_1/2_ =  ∼45 min). This kinetics was slowed down by the presence of E2, requiring up to 4 hrs to be completed (t_1/2_ =  ∼80 min). This result is consistent with a chaperone-like role played by E2 whose co-expression delays folding kinetics. However, the establishment of a DTT-resistant conformation for the E1 protein was a slow process proceeding independently of the presence or absence of E2 and it required approximately 4 hours to be achieved. Indeed, the percentage of the DTT resistant species was roughly equivalent in the two samples, without any yield increase of fully oxidized E1 when co-expressed with E2. Our folding analysis was also extended to detect association of E1 with ER resident chaperones, however we were not able to identify these in our system. In particular, we were unable to reveal any transient association with ER chaperones and calnexin (data not shown). This observation was particularly surprising since in a previous study we were able to detect a transient association of this lectin-type chaperone with E1 expressed as the polyprotein [8]. It could be possible that the accelerated oxidation of unescorted E1 does not require the association with calnexin or that their interaction is so accelerated that it is difficult to detect.

Although our primary focus was E1, we performed parallel analysis of individually expressed E2 folding. To this end, we used a series of monoclonal conformational antibodies that limited our study to the detection of partial or fully folded protein. In our hands, the oxidation pathway of individually expressed E2 was severely impaired as was the achievement of a DTT-resistant conformation. These observations confirm previous studies showing the requirement of E1 co-expression and are consistent with a chaperone-like role of E1 [8], [40]. Recent work from Krey et al. further supports our conclusion on the relative role of E1 and E2 on their own folding [36]. These authors obtained very interesting findings on the overall E2 structure by analysis of a purified soluble truncated form of E2 whose folding was assisted by co-expression of the full length E1.

The finding that individually expressed E1 was able to successfully achieve a complete folding pathway is consistent with assessed data on analogous proteins from related viruses, namely TBE preM or p62 from SFV. However, such proteins have been followed only for the time required to complete folding, which is 60 and 15 min post-synthesis, respectively [37], [38]. Our analysis, however, was extended to determine the stability of unescorted E1 inside the cell. This study revealed that the persistence of intracellular E1 was linked to the contemporary presence of E2, likely associated in a heterodimer. Since inhibition of mannose trimming stabilizes the substrate protein that is targeted for degradation, the recovery of a low amount of E1 in overnight kifunesine-treated cells suggests that endoplasmic reticulum associated degradation (ERAD), with the proteasome as a final destination, are involved in this clearing [41], [42]. Contrary to what was observed for the E1 folding process, E2 does play a role in stabilizing the native E1 conformation.

Overall, E1 and E2 appear to assist each other but via two different strategies. E1 is required for a correct oxidation pathway of E2, slowing down the oxidative kinetics, increasing yield and preventing aggregation for the time required to install productive intra-chain interactions and reshuffling of disulfide bonds. With respect to this aspect, E1 has a similar mechanism of action to that of chaperones belonging to the Hsp70 or the lectin-like family [43], [44]. On the other hands, E2 also slows down oxidation of E1 but it does not appear to interfere with the achievement of the mature conformation of E1 while protecting this molecule from cellular degradation. This behavior mimics the action of species belonging to the Hsp90 family that induce conformational changes of native species leading to their activation or stabilization [45]. According to these conclusions, E2 would always require E1 co-expression in *cis,* whereas a portion of E2 might be sufficient to stabilize full length E1 in a native conformation.

E2 carries the major antigenic epitopes and, for obvious reasons, it has been targeted as the most promising candidate for the development of a protective vaccine [18]. E1 is the less studied molecule of the pair even though it could be an attractive vaccine candidate, in terms of sequence conservation, absence of hypervariable regions and the presence of monoclonal neutralizing anti-E1 antibodies [22], [23]. We believe that this report provides the experimental basis for generating a stable E1 molecule to be purified and investigated for its immunogenic properties.

## Materials and Methods

### Antibodies and reagents

The anti-E2 mAb CBH-7, which recognizes a conformational epitope of E2, and the anti-E1 mAb CBH-111, which recognizes a linear epitope of E1, have been previously described [22], [27]. Endoglycosidase H, complete protease inhibitor EDTA-free tablets, PMSF, NEM and DTT were purchased from Roche; kifunesine was purchased from Sigma-Aldrich.

### Generation of stably transfected cells

A replicon containing the entire genome of HCV genotype 1b, subtype ConI was isolated as previously described [25]. Sequences coding for HCV structural proteins E1 (aa. 165–398), E2p7 (aa. 365–809) and E1E2p7 (aa. 165–809) were amplified from the replicon. Primers used for amplification are:

for E1 5′ CGGCGTCGACATGGCAACAGGGAATCTGCCCGG 3′

5′ GGCCAAAAACACCCTCGGGTAAGAATTCTTTT 3′

for E2p7 5′ CTACGTCGACATGGGGGAACTGGGCTAAGG 3′

5′ CCACGAGCATACGCCTAAGAATTCTTTT 3′

for E1E2p7 5′ CGGCGTCGACATGGCAACAGGGAATCTGCCCGG 3′

5′ GGCCAAAAACACCCTCGGGTAAGAATTCTTTT 3′

Amplified fragments were digested with SalI and EcoRI (New England Biolabs) and cloned into the pCMVIII vector (Chiron corporation) carrying the genticin resistance gene and the dihydrofolate reductase (DHFR) gene under control of an EMCV promoter. The sequences of the inserts were verified by sequencing. In these constructs, all proteins are targeted to the endoplasmic reticulum by their own signal sequence. For our experiments, an E2p7 constructs including a shorter signal sequence (365–383 instead of 352–383) was chosen since, from preliminary experiments, it was found equally efficient in targeting E2p7 to the ER while an increased cellular expression level was obtained in stable transfected cells.

A DHFR-deficientChinese Hamster Ovary (CHO, clone DG44) cells were transfected with constructs expressing E1 (CHO-E1), E2p7 (CHO-E2p7) or E1E2p7 (CHO-E1E2p7), and selected for stable clones as described previously [46]. Screening of HCV proteins expressing clones was performed by Western blot for the presence of recombinant proteins. Cells expressing E1, E2p7 or E1E2p7 were further expanded and grown in α-MEM without nucleoside and ribonucleosides, supplemented with 10% dialyzed fetal bovine serum (dFBS, Gibco-Invitrogen), 1 mM L-glutamine, 0.02% proline and antibiotics, 100 µM geneticin (G-418, Gibco-Invitrogen) at 37°C with 5% CO_2_.

Untransfected cells were grown in F12 (Gibco-Invitrogen) supplemented with 10% dFBS, 1 mM L-glutamine, 0.02% proline and antibiotics, at 37°C with 5% CO_2_.

### Cell labeling and pulse-chase analysis

Metabolic labeling was performed by adding 100–200 µCi/ml of [^35^S]-labeled cysteine and methionine (ProMix, Amersham Pharmacia Biotech) to cell medium and incubating for 6 or more hrs at 37°C with 5% CO_2_. For pulse-chase experiments, subconfluent CHO-E1, CHO-E2p7 and CHO-E1E2p7 cells grown in 60 mm diameter dishes (Falcon) were starved for 90 min in cysteine- and methionine-free α-MEM (starving medium). Cells were then pulsed supplementing the cysteine- and methionine-free medium with 800 µCi/ml of [^35^S]-labeled cysteine and methionine (ProMix, Amersham Pharmacia Biotech) for 20 min. After washing cells once with starving medium, the chase-period was started by adding α-MEM supplemented with 25 mM unlabeled cysteine and methionine. For DTT resistance experiments, the chase-time was followed by 5 min incubation in presence of 5 mM DTT added to the medium. Experiments with the ER mannosidase inhibitor were performed by supplementing culture medium with 20 µM kifunesine during pulse- and chase-time.

At the end of each chase-time, cells were placed on ice for 5 min in cold PBS containing 1 mM PMSF and 20 mM NEM. After removal of cold PBS, 1 ml of lysis buffer (50 mM Hepes pH 6.8, 200 mM NaCl, 1 mM EDTA, 1% Triton X-100, 1 mM PMSF, 20 mM NEM and complete protease inhibitor EDTA-free) was added to each plate and cells were removed using a cell scraper. The suspensions were then transferred to a 1.5 ml eppendorf tube and incubated for 30 minutes at 4°C on a rotating wheel. Total lysates were centrifuged for 30 min at 16000 x g at 4°C. Post-nuclear supernatants (PNSs) were used for immunoprecipitation experiments.

### Immunoprecipitation, SDS-PAGE and DTT-resistance analysis

PNSs were precleared with protein G and protein A coupled to Sepharose Fast Flow (Amersham Pharmacia Biotech) for 1 hr at 4°C. When specified, we determined the fraction of labeled amino acids incorporated into proteins by thricholroacetic (TCA) precipitation procedure. Briefly, 2 µl of each precleared lysate (triplicates) were added to 200 µl of 20% cold thrichloroacetic acid, mixed and placed on ice for 10 min to allow precipitation of high molecular weight molecules. Each precipitate was collected via vacuum filtration through a Whatmann GF/C glass fiber filter prewetted with 5% TCA. After three washes of the tube with cold 5% TCA passed through the filter, dryed filters were placed in scintillation vials to which 2 ml of aqueous scintillation cocktail were added and count in a scintillation counter. The cpm obtained reflect the amount of radioactivity that was incorporated into proteins. Equivalent amounts of total labeled proteins were used for immunoprecipitations. Immunoprecipitations were carried out overnight with 0.5 µg CBH-7 and 0.5 µg CBH-111 at 4°C in presence of 6 µl of Dynabeads Protein G (Dynal Biotech ASA).

The beads containing the immunoprecipitated proteins were collected with a magnetic support (Biorad) and washed 3 times with cold lysis buffer containing 0.5% Triton. Elution of the proteins from the beads was performed with SDS loading buffer at 95°C for 5 min. Samples were then analyzed by SDS-PAGE, followed by autoradiography (Storage Phosphor Screen, Amersham). For SDS-PAGE in reducing condition, a final concentration of 250 mM DTT was added to the samples.

For DTT-resistance analysis, duplicate plates were prepared for each sample. At each chase-time, 5 mM DTT was added in the culture medium to one plate of the pair 5 min before the end of the chase-period. Following cell lysis and immunoprecipitation, SDS-PAGE analysis was then performed in both non-reducing and reducing conditions.

### Endoglycosidase digestion

Endoglycosidase H digestions were performed on aliquots (10 µl) of immunoprecipitated proteins recovered from the beads in SDS loading buffer. To quench the SDS, samples were added to 200 ml (20 times v/v) of EndoH buffer (125 mM Sodium Acetate pH 5.8, 250 mM β-mercaptoethanol, 5 mM PMSF) containing 1% triton X-100. After warming at 95°C for 3 min, samples were allowed to reach room temperature and incubated with 10 mU endoglycosidase H. The reactions were performed at 37°C overnight. Digested proteins were precipitated in TCA and resuspended in loading buffer with 250 mM DTT for SDS-PAGE analysis.
